# Beneficial effects of upadacitinib on a subgroup of patients with atopic dermatitis and hidradenitis suppurativa: a multicenter, real-world retrospective study

**DOI:** 10.3389/fimmu.2026.1770969

**Published:** 2026-02-25

**Authors:** F. Russo, N. Schettini, G. L. Scaglione, N. Gori, F. Manzo Margiotta, E. Del Duca, T. Bianchelli, L. Calabrese, E. Antonelli, F. Giuliani, M. Corbeddu, M. Galluzzo

**Affiliations:** 1 Dermatological Department, Istituto Dermopatico dell’Immacolata, Istituto Dermopatico dell'Immacolata - Istituto Scientifico di Ricerca, Ricovero e Cura (IDI-IRCCS), Dermatological Research Hospital, Rome, Italy; 2 Section of Dermatology, Department of Medical Sciences, University of Ferrara, Ferrara, Italy; 3 Bioinformatics Unit, Istituto Dermopatico dell’Immacolata, Istituto Dermopatico dell'Immacolata - Istituto Scientifico di Ricerca, Ricovero e Cura (IDI-IRCCS), Dermatological Research Hospital, Rome, Italy; 4 Dermatologia, Dipartimento Universitario di Medicina e Chirurgia Traslazionale, Università Cattolica del Sacro Cuore, Rome, Italy; 5 Complex Operating Unit (U.O.C.) Dermatologia, Dipartimento di Scienze Mediche e Chirurgiche, Fondazione Policlinico Universitario A. Gemelli IRCCS, Rome, Italy; 6 Department of Dermatology, University of Pisa, Pisa, Italy; 7 Health Science Interdisciplinary Center, Sant’Anna School of Advanced Studies, Pisa, Italy; 8 Dermatology Unit, Department Of Medical And Cardiovascular Sciences, Sapienza University of Rome, Rome, Italy; 9 Dermatology Unit, Istituto Nazionale di Riposo e Cura per Anziani, Istituto Nazionale di Ricovero e Cura degli Anziani (INRCA)-IRCCS Hospital, Ancona, Italy; 10 Dermatology Unit, Department of Medical, Surgical and Neurological Sciences, University of Siena, Siena, Italy; 11 Dermatology Section, Department of Medicine and Surgery, University of Perugia, Perugia, Italy; 12 Dermatology Unit, Polyclinic Hospital “SS Annunziata”, Department of Medicine and Aging Sciences, University “G. D’Annunzio”, Chieti, Italy; 13 Dermatology Clinic, Department of Medical Sciences and Public Health, University of Cagliari, Cagliari, Italy; 14 Dermatology Unit, Fondazione Policlinico “Tor Vergata”, Rome, Italy; 15 Department of Systems Medicine, University of Rome “Tor Vergata”, Rome, Italy

**Keywords:** atopic dermatitis, comorbidity, hidradenitis, therapy, upadacitinib

## Abstract

**Introduction:**

Although AD and HS are significantly different clinically, they share common systemic comorbidities and some immunological pathways. The aim of this multicentric Italian retrospective study is to assess therapeutic effects of upadacitinib on concomitant HS in AD patients.

**Materials and methods:**

Multicentric, observational, real-life retrospective study on 195 patients with moderate to severe AD treated with upadacitinib. Specifically, a subgroup analysis of AD+HS patients was conducted. Demographic, clinical data, outcome measures (EASI, NRS itch, sleep and pain, DLQI POEM and ADCT) were collected at baseline (week 0) and at subsequent time points (week 4, week 16, week 32, and week 52). Additionally, IHS4 IHS4–55 and HiSCR were the tools used to evaluate the clinical course of HS in the AD+HS subgroup.

**Results:**

A total of 195 AD patients were included, 7 (3,6%) patients also suffered of concomitant HS. In the AD+HS subgroup, mean IHS4 decreased from 6.9 ± 4.3 at baseline to 2.2 ± 1.8 at week 16 and 0.2 ± 0.5 at week 32. Reductions of mean baseline severity of HS tool (IHS4) score was detected as early as after only 4 weeks of treatment. Accordingly, HiSCR and HIS4-S5 were achieved by 4/7 patients at week 4, 5/6 at week 16, and 4/4 at week 32.

**Discussion:**

In the AD+HS subgroup, improvements were observed for both AD and HS-specific outcomes. Our data, although preliminary, show that upadacitinib could be a valid therapeutic choice in the treatment of patients with AD and concomitant HS.

## 1 Introduction

Atopic dermatitis (AD) is a chronic inflammatory skin disease characterized by intense itching and eczematous lesions. It affects 15% to 25% of children and 4% to 7% of adults and usually appears within the first 5 years of childhood (1). Hidradenitis suppurativa (HS) is a chronic inflammatory disease with recurrent course affecting skin-bearing apocrine glands, typically in the axillae, breasts, groin, and perineum. HS affects approximately 1% of the general population and typically begins post puberty, with the highest incidence observed among young women aged 20 to 29 (2).

The coexistence of HS and AD has already been reported in the literature (3, 4). Epidemiological studies have shown the presence of systemic comorbidities common to both pathologies (e.g. hypertension, obesity and psychiatric disorders) (2, 5). Common risk factors between AD and HS include smoking, female sex, and African-American race (4).

Both diseases have a pathogenesis that involves the complex interaction between immune dysregulation, environmental triggers, genetic factors and dysbiosis (6). It was previously believed that AD and HS are driven by different immunologic pathways. However, the recent awareness of a broader Th1, Th2 and Th17 dysregulation in AD, which goes beyond the T helper (Th2) cell-driven disease, has highlighted common immunological pathways with HS, driven by Th1/Th17 cells (5). Furthermore, similar deficient notch signaling (that plays a key role in the regulation of epidermis and pilosebaceous unit differentiation) and antimicrobial peptide (AMP) dysregulation were seen in both conditions (4). While in HS the role of Notch signaling dysregulation is unclear, in AD it can cause epidermal barrier defects and an increase in pro-inflammatory cytokines, such as thymic stromal lymphopoietin (TSLP) (7). Although the role of AMP dysregulation remains to be fully elucidated, in both AD and HS it acts by promoting an inflammatory response in the context of altered skin flora (8). Moreover, the role of other shared biomarkers, such as interleukin-13 or periostin, which promote fibrosis in the pathogenesis of both AD and HS, remains to be clarified (5). A better understanding of these shared immune pathways could guide the appropriate use of immunomodulatory drugs to avoid exacerbating one disease to the benefit of the other.

Upadacitinib, a JAK-1 inhibitor (JAKi), has demonstrated remarkable efficacy in the treatment of moderate to severe AD (9) and is increasingly being used and studied across a broad spectrum of dermatological disorders including HS. However, published clinical data on the specific use of upadacitinib for HS are very limited and several clinical trials are currently ongoing. The potential therapeutic targets for this JAKi are proinflammatory cytokine such as IL-6, IL-23, and interferons which have been shown to be upregulated in sites affected by HS (10).

The aim of this multicentric Italian retrospective study is to assess therapeutic effects of upadacitinib on concomitant HS in AD patients.

## 2 Patients and methods

### 2.1 Study design and patients

We conducted a multicentric, observational, real-life retrospective study on 195 patients with moderate to severe AD treated with upadacitinib in the Dermatology section of eleven Italian center from march 2024 to march 2025. In this study we also retrospectively assessed therapeutic effects of upadacitinib on concomitant HS in AD patients.

Inclusion criteria were age ≥ 18 years old and the presence of moderate to severe AD for at least 6 months. Demographic and clinical data, including sex, age, body mass index (BMI), age at disease onset, disease duration, clinical phenotype, smoking status, personal and family history of atopic and non-atopic comorbidities, previous therapies, and baseline total serum IgE levels, were sourced from medical records and collated in a dedicated database. HS diagnosis was retrieved from routine medical-record documentation (no systematic screening), and HS severity scores were calculated at each visit. For patients with available data, outcome measures were collected at baseline (week 0) and at subsequent time points (week 4, week 16, week 32, and week 52). These scores included EASI (Eczema Area and Severity Index), NRS (Numeric Rating Scale) for itch, sleep and pain, DLQI (Dermatology Life Quality Index), POEM (Patient-Oriented Eczema Measure), and ADCT (Atopic Dermatitis Control Tool) for AD. Additionally, IHS4 (International Hidradenitis Suppurativa Severity Score System), IHS4–55 and HiSCR (Hidradenitis Suppurativa Clinical Response) were the tools used to evaluate the clinical course of HS. IHS4 represents a dynamic scoring system assessing disease severity based on the number of HS characteristic inflammatory lesions multiplied by a specific coefficient equivalent to 1 for nodules, 2 for abscesses and 4 for draining tunnels. The disease can be classified as mild, moderate or severe depending on the sum of the individual values (11). IHS4–55 is a dichotomous outcome that is achieved in the presence of at least 55% reduction in IHS4 score compared to the baseline. It represents a validated tool to assess treatment efficacy in HS patients (12). HiSCR delivers the same outcome as IHS4-55, but it is achieved where there is a reduction of at least 50% in the sum of nodules and abscesses and in the absence of an increasing number of abscesses and draining tunnels (13).

### 2.2 Statistical analysis

Continuous variables are summarized as mean and standard deviation (SD), and categorical variables as counts and percentages. Longitudinal changes in outcome measures for the whole AD population and for the AD+HS subgroup were summarized using mean ± SD for continuous variables and n/total (percentage) for binary endpoints.

For the overall AD population, comparisons between baseline and subsequent time points were performed using the Wilcoxon signed-rank test for paired continuous variables. Repeated measures were summarized at each scheduled visit; no longitudinal mixed-effects models were fitted. For the AD+HS subgroup, HS outcomes were summarized descriptively due to the very small sample size and variable availability of HS assessments across visits. All analyses were performed using R (version 4.3.3) and relevant packages (dplyr, gtsummary, flextable, officer, etc).

## 3 Results

### 3.1 Results in the overall AD population

A total of 195 patients (83 females and 112 males) were included in the analysis (**Table 1**). The mean age was 35.4 ± 13.9 years, the mean AD onset age was 12.9 ± 18.1 years, while BMI was 24.8 ± 3.9. The mean disease duration was 23.0 ± 12.9 years. The most frequent clinical phenotypes were diffuse (34%), head and neck (26%) and flexural (18%). We found that 29% of patients currently smoke and 5.3% had recently stopped smoking. A family history of atopy was reported in 33% of cases: 55%, 35% and 31% of patients had a positive personal history for allergic rhinitis, allergic conjunctivitis and asthma, respectively. Demographic, clinical characteristics and previous systemic therapies of the overall AD population were reported in **Table 1**. Based on medical history and/or clinical examination, no patients showed contraindications to the use of upadacitinib. The majority of patients (63.9%) started treatment with upadacitinib at a dosage of 15 mg/day, while 36.1% started with 30 mg/day. The dosage was decreased or increased during treatment, due to poor response, side effects or prolonged disease remission.

**Table 1:** Demographic and clinical characteristics of overall AD population.

Characteristic	N = 195^1^
Sex	
Male	112 (57%)
Female	83 (43%)
Age (years)	35.4 (13.9)
BMI	24.8 (3.9)
Age at disease onset	12.9 (18.1)
Disease duration (years)	23.0 (12.9)
Clinical phenotype	
Diffuse eczema	65 (34%)
Diffuse eczema, chronic hand eczema	1 (0.5%)
Chronic hand eczema	5 (2.6%)
Erythrodermic eczema	4 (2.1%)
Flexural	34 (18%)
Flexural, diffuse eczema	2 (1.0%)
Flexural, head and neck eczema	3 (1.6%)
Follicular	2 (1.0%)
Head and neck eczema	51 (26%)
Head and neck eczema, diffuse eczema	2 (1.0%)
Nummular eczema	5 (2.6%)
Nummular, follicular, psoriasiform eczema	1 (0.5%)
Prurigo nodularis	5 (2.6%)
Psoriasiform eczema	12 (6.2%)
Psoriasiform eczema, head and neck eczema	1 (0.5%)
Smoking status	
Absent	124 (66%)
Previous	10 (5.3%)
Current	54 (29%)
Atopic comorbidities	
Allergic asthma	50 (31%)
Allergic rhinitis	89 (55%)
Allergic conjunctivitis	55 (35%)
Nasal polyposis	2 (1.0%)
Food allergy	32 (17%)
Eosinophilic esophagitis	1 (0.5%)
Non atopic comorbidities	
Alopecia areata	8 (4.1%)
Psoriasis	4 (2.1%)
ACD*	4 (2.1%)
Rheumatoid arthritis	2 (1.0%)
Chronic inflammatory bowel diseases	3 (1.5%)
Systemic comorbidities	
Hypertension	15 (7.7%)
Diabetes	3 (1.5%)
Dyslipidemia	19 (9.8%)
Cardiovascular diseases	3 (1.5%)
Ocular diseases	9 (5.4%)
Family history of atopy	
No	119 (67%)
Yes	58 (33%)
Previous systemic therapies	
Steroids	113 (65%)
Cyclosporine	96 (53%)
Dupilumab	71 (41%)
Tralokinumab	22 (11%)
Lebrikizumab	0 (0%)
Abrocitinib	3 (1.5%)
Baricitinib	6 (3.1%)

^1^n (%); Mean (SD); *Allergic contact dermatitis.

**Table 1**. Demographic and clinical characteristics were summarized using means and standard deviations (SD) for continuous variables, and absolute frequencies with percentages for categorical variables.

**Figure 1:**
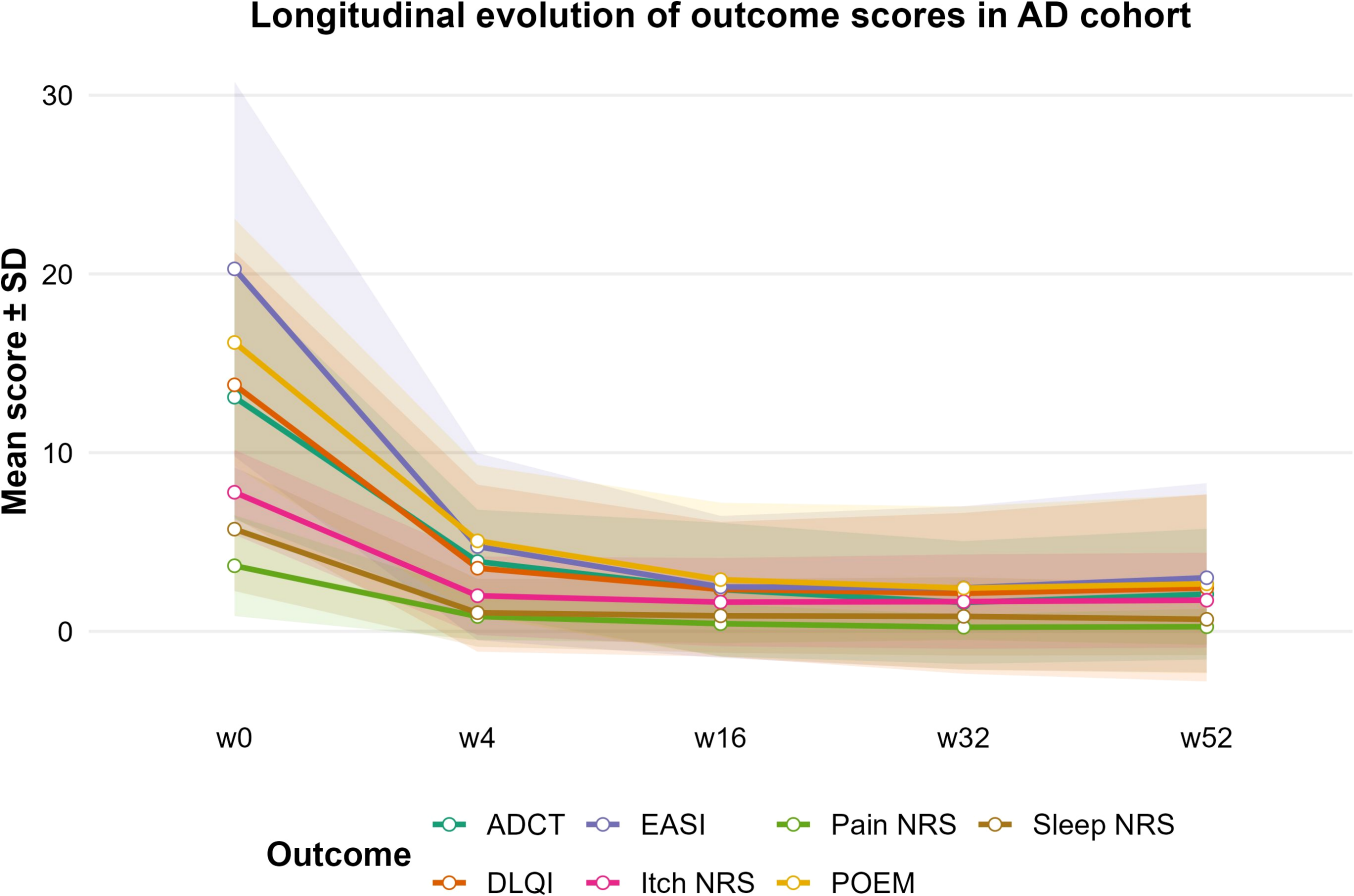
Longitudinal evolution of outcome scores in the overall atopic dermatitis (AD) cohort treated with upadacitinib. Lines represent mean values at baseline (week 0) and follow-up visits (weeks 4, 16, 32, and 52) for EASI, ADCT, DLQI, POEM, and Numerical Rating Scale (NRS) for itch, sleep, and pain. Shaded areas indicate ±1 standard deviation (SD).

In the overall AD population, all clinical outcomes improved from baseline to subsequent follow-up visits (**Table 2**; **Figure 1**). Mean EASI score decreased from 20.3 ± 10.5 at baseline to 2.5 ± 4.0 at week 16 and 3.0. ± 5.3 at week 52. Similar trends were observed for the Numerical Rating Scale (NRS) for itch, sleep, and pain, as well as DLQI, POEM, and ADCT. The longitudinal trends of these outcomes are illustrated in **Figure 1**, where the mean value of each score is plotted over time with shaded areas representing ±1 standard deviation.

**Table 2:** Longitudinal outcome measures (mean ± SD).

Variable	w0	w4	w16	w32	w52
EASI	20.3 ± 10.5	4.7 ± 5.2	2.5 ± 4.0	2.4 ± 4.6	3.0 ± 5.3
NRS itch	7.8 ± 2.4	2.0 ± 2.2	1.6 ± 2.5	1.7 ± 2.6	1.7 ± 2.7
NRS sleep	5.7 ± 3.5	1.0 ± 1.9	0.9 ± 2.1	0.8 ± 2.2	0.7 ± 2.0
NRS pain	3.7 ± 2.8	0.8 ± 1.3	0.4 ± 1.1	0.2 ± 0.7	0.3 ± 1.0
DLQI	13.8 ± 7.4	3.5 ± 4.7	2.4 ± 3.8	2.1 ± 4.5	2.4 ± 5.2
POEM	16.2 ± 6.9	5.1 ± 4.2	2.9 ± 4.3	2.4 ± 4.6	2.7 ± 5.0
ADCT	13.1 ± 6.8	3.9 ± 2.9	2.3 ± 3.7	1.6 ± 3.4	2.1 ± 3.7

**Table 2**. Repeated measures were summarized at each scheduled visit (baseline, weeks 4, 16, 32, 52). Continuous outcomes are reported as mean ± SD.

### 3.2 Results in the AD+HS subgroup

Of 195 AD patients treated with upadacitinib, 7 also suffered from concomitant HS (Demographic, clinical characteristics were reported in **Table 3**). In this subgroup, mean age was 26.0 ± 7.4 years, mean disease duration was 20.3 ± 6.2 years, and 4/7 were male with a mean BMI of 28.5 ± 4.3. No one had a family history of atopy. In the AD+HS subgroup, 4/7 patients received upadacitinib 15 mg/day and 3 out 7 received 30 mg/day. No dose variation occurred in the AD+HS patient subgroup.

**Table 3:** Demographic and clinical characteristics of patients with AD and concomitant HS were summarized using means and standard deviations (SD) for continuous variables, and absolute frequencies with percentages for categorical variables.

Characteristic	N = 7^1^
Sex	
Male	4 (57%)
Female	3 (43%)
Age (years)	26.0 (7.4)
BMI	28.5 (4.3)
Age at disease onset	11.7 (13.3)
Disease duration (years)	20.3 (6.2)
Clinical phenotype	
Diffuse eczema	3 (43%)
Flexural	3 (43%)
Head and neck eczema	1 (14%)
Smoking status	
Absent	5 (71%)
Previous	0 (0%)
Current	2 (29%)
Atopic comorbidities	
Allergic rhinitis	3 (43%)
Allergic conjunctivitis	2 (29%)
Family history of atopy	
No	7 (100%)
Yes	0 (0%)
Previous systemic therapies	
Steroids	6 (86%)
Cyclosporine	2 (29%)
Dupilumab, Tralokinumab, Lebrikizumab, Abrocitinib, Baricitinib	0 (0%)

^1^n (%); Mean (SD)

In the AD+HS subgroup, improvements were observed for HS-specific outcomes (**Table 4**). Mean IHS4 decreased from 6.9 ± 4.3 at baseline (n=7) to 3.1 ± 2.3 at week 4 (n=7), 2.2 ± 1.8 at week 16 (n=6) and 0.2 ± 0.5 at week 32 (n=4). Across follow-up visits, HiSCR and HIS4-S5 were met by 4/7 patients at week 4, 5/6 at week 16, and 4/4 at week 32; denominators reflect available assessments at each time point. The decreasing denominators in the AD+HS subgroup reflect differences in treatment initiation dates and, consequently, heterogeneous observational follow-up windows in this retrospective real-world cohort.

**Table 4:** HS outcome measures in the AD+HS subgroup (mean ± SD or n/N).

Variable	w0 (n=7)	w4 (n=7)	w16 (n=6)	w32 (n=4)
HS IHS4	6.9 ± 4.3	3.1 ± 2.3	2.2 ± 1.8	0.2 ± 0.5
HS HiSCR (responders, n/N)	-	4/7	5/6	4/4
HS IHS4-55 (responders, n/N)	-	4/7	5/6	4/4

**Table 4**. HS outcomes were summarized descriptively at each scheduled visit. Continuous outcomes are reported as mean ± SD; responder outcomes are reported as n/N.

## 4 Discussion

Although AD and HS are significantly different clinically (onset, pathogenesis, and treatment), they share common systemic comorbidities and some immunological pathways. Additionally, both diseases are associated with a deep impact on quality of life and an increased burden of health care use (14). Sherman et al. in a large epidemiological study showed a statistically significant bidirectional association between AD and HS (1). Specifically, patients with HS and comorbid AD were more likely to be younger, female and non-smokers and have a lower BMI than patients with HS alone.

Upadacitinib represents an effective and safe option for the treatment of moderate to severe AD as shown in clinical trials and real-world clinical practice on large cohorts of patients. In an Italian observational study on one hundred and forty-six patients followed for 48 weeks, the achievement of EASI 75, EASI 90 and EASI 100 was observed in 87.6%, 69.1% and 44.3% of the sample, respectively (15). Studies on Japanese patients showed similar results both on AD clinical signs and symptoms even though with lower percentages of EASI 75, EASI 90 and EASI 100 improvement (16). Ibba et al., in a recent systematic review including 17 real-world studies, reported high efficacy of upadacitinib across diverse patient phenotypes, with no significant new safety concerns (17). In AD patients with an insufficient response to 15 mg, a dose increase of upadacitinib up to 30 mg increase significantly eczematous rash and pruritus. The treatment responsiveness to upadacitinib 30 mg appeared lower on the head and neck compared with other body sites, particularly the trunk, but remain a valid option for patients with a limited response to 15 mg treatment (18).

The broad-spectrum inhibition of many pro-inflammatory cytokines has led to the evaluation of upadacitinib in other pathological conditions, including HS. In a phase 2 study, patients with moderate-to-severe HS treated with upadacitinib 30 mg have experienced clinical improvement with a greater HiSCR50 at week 12 when compared with the placebo group (38.3% vs 23.8%) (19). A phase 3 study (NCT05889182) is still ongoing.

**Figure 2:**
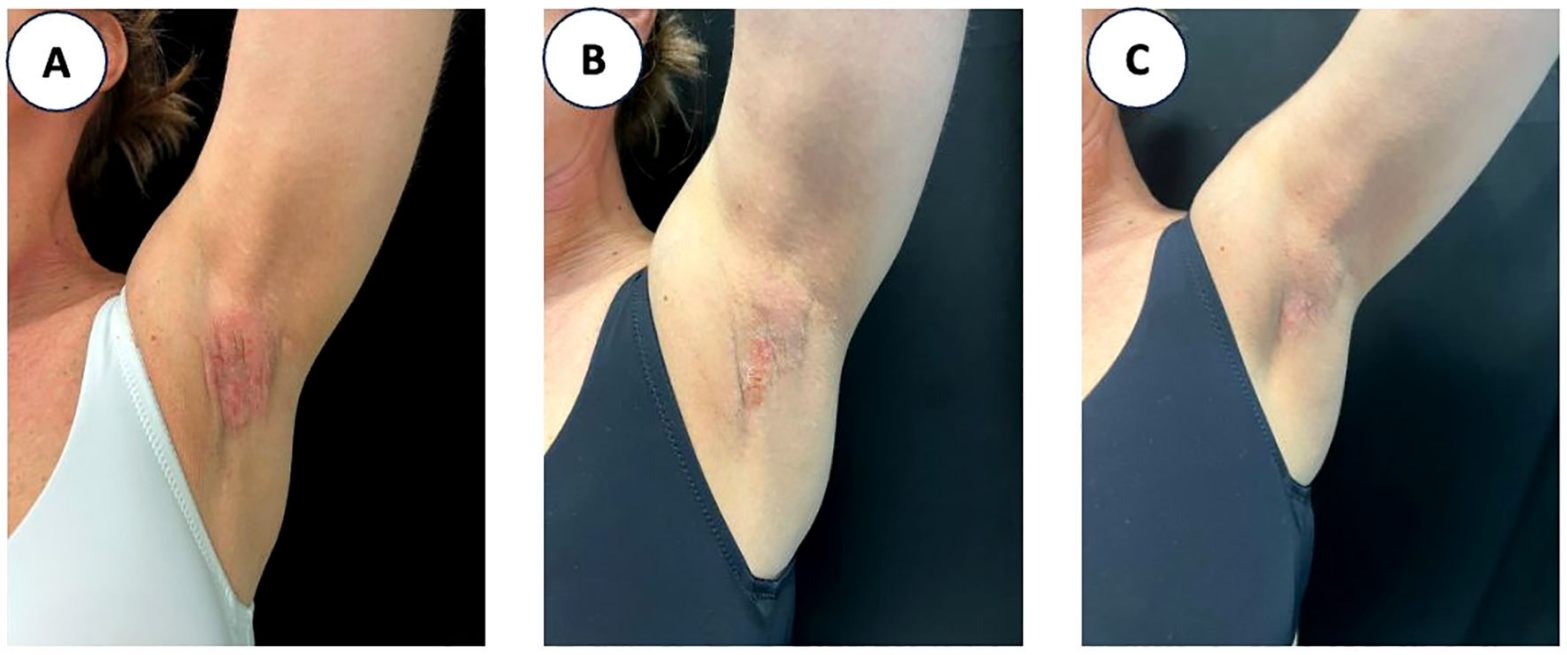
Clinical images of a female patient with concomitant atopic dermatitis (AD) and hidradenitis suppurativa (HS) treated with upadacitinib. Improvement of left axillary lesions is shown at baseline [week 0, **(A)**], week 16 **(B)**, and week 52 **(C)**. Nodules and erythema progressively reduced in number and intensity, reaching complete clearance of the skin at week 52.

In our cohort, AD clinical outcomes improved from baseline to week 52, in line with data reported in clinical trials and real-life experience. In the AD+HS subgroup, improvements were observed in both AD and HS outcomes. In this subset, 4/7 patients received upadacitinib 15 mg/day and 3/7 received 30 mg/day. Mean IHS4 score decreased from 6.9 ± 4.3 at baseline to 3.1 ± 2.3 at week 4, with further decreases over time (2.2 ± 1.8 at week 16 and 0.2 ± 0.5 at week 32), with HiSCR and IHS4–55 responses observed at each visit among patients with available assessments (**Figure 2**; **Table 4**); denominators decreased over time (n=7, 6, and 4). Accordingly, HiSCR was achieved by 4/7 (57.1%) at week 4 and by 4/4 (100%) at week 32 among patients assessed at those visits. Our observations are consistent with the phase 2 study on the efficacy of upadacitinib in HS where 34% of patients achieved HiSCR50 at 4 weeks and 38.3% at week 12 (19). Such early clinical improvements confirm the rapidity of action of upadacitinib in immune-mediated diseases, such as rapid pain relief in psoriatic arthritis and ankylosing spondylitis (20). In a retrospective cohort study on 20 HS patients treated with Upadacitinib, HiSCR50 was achieved in 75% of subjects at week 4 and in 100% at week 12 with maintenance of clinical response at week 24. DLQI scores and pain rating scores improved as well (21). A proteomic analysis on this cohort showed a significant reduction in the expression of inflammatory cytokines such as IL-12, IL-23 and IL-10 by week 4 of treatment. In clinical responder patients, a rapid attenuation of B-Cell and IFN-γ associated chemokines was observed (22). This broad-spectrum inhibition could be an extremely useful therapeutic weapon in HS, whose pathogenesis is linked to the simultaneous activation of innate and adaptive immune mechanisms, in part driven by JAK-STAT signaling-dependent cytokines (23, 24). Studies published to date suggest that upadacitinib is one of the most promising small molecules for the treatment of HS (25, 26).

In our study the favorable trajectories observed may be related to the predominance of moderate HS forms, with fewer lesions and fewer drainage tunnels, potentially reflecting a lower inflammatory burden compared with clinical trials. Nevertheless, given that HS is characterized by the formation of drainage tunnels and scarring, timely treatment may be relevant even in moderate forms, as delayed diagnosis and management can compromise the response to biologics (27). Furthermore, the presence of patients who had not previously received HS-targeted biologics (TNFalpha or IL-17 inhibitors), but only antibiotic therapies, may have contributed to the observed clinical outcome. HS is a condition with an increased risk of infections regardless of the treatment used to manage it. Meta-analysis studies showed an incidence of 24.2% with a greater presence of respiratory and skin infections. However severe infections were rare (28). In the phase 2 randomized, placebo-controlled study, at 48 weeks no severe infections were observed in HS patients treated with upadacitinib 15 mg, while among patients who received 30 mg, only 2 out of 47 had severe infections. Anyway, the only adverse event potentially related to the underlying disease and treatment was a case of severe inguinal cellulitis (19). On the other hand, atopic dermatitis also presents an increased intrinsic risk of infections related to barrier defects and a reduction in antimicrobial peptides (29). However, the use of upadacitinib, as highlighted by clinical studies, did not lead to an increased risk of severe infections (30). Obviously, the risk of infection in patients with AD and concomitant HS must always be considered. The possible appearance of signs and symptoms of infection must be adequately treated with specific antibiotic therapy, temporarily suspending or otherwise adjusting upadacitinib until the infection has resolved. No rescue strategies were used in our patients, probably due to the presence of moderate clinical manifestations of HS and the rapid resolution of the lesions.

This study has some limitations, including the small size of the AD+HS subgroup and the retrospective design. HS identification and severity assessments were based on routine medical records (not systematic screening), and follow-up completeness varied across visits. In such a small subgroup, decreasing denominators may influence the interpretation of observed patterns over time and limit the generalizability of responders patterns. Additionally, no adjustment for potential confounders (e.g., baseline HS severity, BMI, smoking status, prior HS treatments, and dose changes) was performed. Further studies with standardized HS assessment and prospective designs are needed to better characterize outcomes in patients with concomitant AD and HS.

## 5 Conclusions

Our data, although preliminary, suggest that upadacitinib could be a valid therapeutic choice in the treatment of patients with AD and concomitant HS. It is essential that dermatologists be aware of this association and perform skin examinations with particular attention to the sites typical of both conditions. Awareness of the potential co-occurrence of HS and AD is crucial for therapeutic choice and patient-centered management.

## Data Availability

The original contributions presented in the study are included in the article/supplementary material. Further inquiries can be directed to the corresponding author.
